# Chloroplast genome of the wild tuber-bearing diploid potato relative *Solanum chacoense*

**DOI:** 10.1080/23802359.2017.1413309

**Published:** 2017-12-07

**Authors:** Kwang-Soo Cho, Jang-Gyu Choi, Ji-Hong Cho, Ju-Sung Im, Young-Eun Park, Su-Young Hong, Tae-Ho Park

**Affiliations:** a Highland Agriculture Research Institute, National Institute of Crop Science, Rural Development Administration, Pyeongchang, South Korea;; b Department of Horticulture, Daegu University, Gyeongsan, South Korea

**Keywords:** Chloroplast, genome, genome sequence, *Solanum chacoense*

## Abstract

*Solanum chacoense* is a wild tuber-bearing species belonging to Solanaceae family. The chloroplast genome of the species was completed by *de novo* assembly using a small amount of whole genome sequencing data. The genome is the circular DNA molecule with a length of 155,532 bp containing 159 predicted genes totally, including 105 protein-coding, 45 tRNA and eight rRNA genes. Maximum-likelihood phylogenetic analysis with 26 species in Solanaceae revealed that *S. chacoense* is the most closely grouped with *S. commersonii*.

*Solanum chacoense* is a wild tuber-bearing species originating from Bolivia and a relative to the cultivated potato, *S. tuberosum* (Hawkes 1990). Because of its importance in several desirable characteristics including resistance to crucial diseases and harmful insects such as late blight (Park et al. 2009), bacterial wilt (Chen et al. 2013, 2016), potato leaf roll virus (Brown and Thomas 1993), common scab (Braun et al. 2017), Colorado potato beetle (Sinden et al. 1980; Molnár et al. 2017), the species has been used for potato breeding. However, the two species are sexually incompatible because of different endosperm balance numbers (EBNs) and ploidy levels of the genomes, with *S. chacoense* and *S. tuberosum* reported as diploid and tetraploid with EBN values of 2 and 4, respectively (Johnston et al. 1980; Ortiz and Ehlenfeldt 1992; Cho et al. 1997). It caused that somatic hybridization of the two species has been attempted for breeding and importance to obtain sequence information of chloroplast in potato breeding program has increased (Chen et al. 2013, 2016; Cho and Park 2016; Molnár et al. 2017)

The *S. chacoense* (PI201846) was provided by Highland Agriculture Research Institute, South Korea. An Illumina paired-end (PE) genomic library was constructed with total genomic DNA according to the PE standard protocol (Illumina, San Diego, CA) and sequenced using an Illumina HiSeq2000 at Macrogen (http://www.macrogen.com/kor/). Low-quality bases with raw scores of 20 or less were removed and approximately 4.7 Gbp of high quality of PE reads were assembled by a CLC genome assembler (CLC Inc, Rarhus, Denmark) (Kim et al. 2015). Principal contigs representing the chloroplast genome were retrieved from the total contigs using Nucmer (Kurtz et al. 2004) with the chloroplast genome sequence of *S. commersonii* (KM489054) as the reference sequence (Cho et al. 2016). The representative chloroplast contigs were arranged in order based on BLASTZ analysis (Schwartz et al. 2003) with the reference sequence and connected to a single draft sequence by joining overlapping terminal sequences. Chloroplast genes were predicted using DOGMA (Wyman et al. 2004) and BLAST searches.

The complete chloroplast genome of *S. chacoense* (GenBank accession no. MF471371) was 155,532 bp in length including 25,592 bp inverted repeats (IRa and IRb) regions separated by small single copy (SSC) region of 18,376 bp and large single-copy (LSC) region of 85,972 bp with the typical quadripartite structure of most plastids, and the structure and gene features were typically identical to those of higher plants. A total of 159 genes with an average size of 582 bp were annotated including 106 protein-coding genes with an average size of 762 bp, 45 tRNA genes and 8 rRNA genes. An overall GC content was 37.89%.

Phylogenetic analysis was performed using chloroplast coding sequences of *S. chacoense* and 25 published species in Solanaceae family by a maximum-likelihood method in MEGA 6.0 (Tamura et al. 2013). According to the phylogenetic tree, *S. chacoense* belonged to the same clade in *Solanum* species as expected, and interestingly, it was most closely grouped with *S. commersonii* (Figure 1).

**Figure 1.**
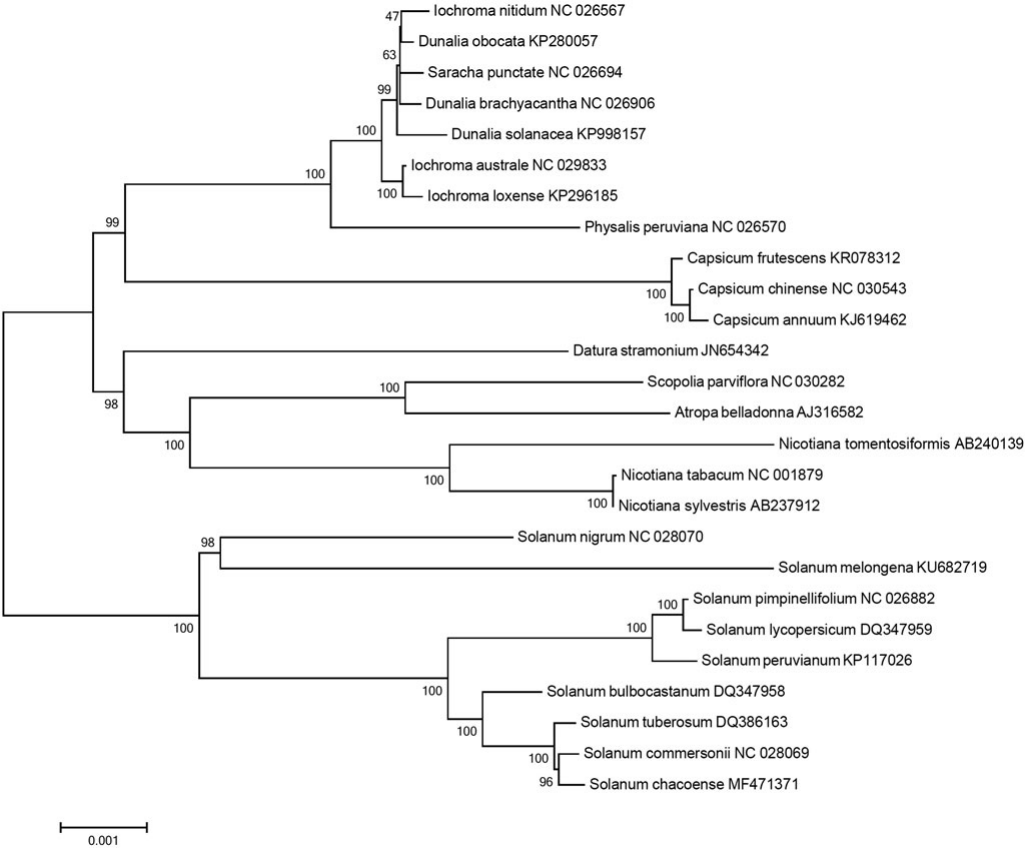
Maximum-likelihood phylogenetic tree of *S. chacoense* with 25 species belonging to the Solanaceae based on chloroplast protein-coding sequences. Numbers in the nodes are the bootstrap values from 1000 replicates.
